# Exploring the needs and coping strategies of family caregivers taking care of dying patients at home: a field study

**DOI:** 10.1186/s12904-023-01315-0

**Published:** 2023-12-12

**Authors:** Xiaotian Zhang, Tianmeng Xu, Yuan Qin, Minghui Wang, Zhaoyu Li, Jingyu Song, Qianqian Tang, Zidan Wang, Lijie Xu, Lingling Wu, Peng Yue

**Affiliations:** 1 School of Nursing, Capital Medical University, 10 Xitoutiao, Youanmen, Fengtai District, Beijing, 100069 China; 2 Wenzhou Medical University, University Town, Chashan, Wenzhou, Zhejiang 325035 China; 3 Hospice & Palliative Care Department Ward of Beijing Haidian Hospital, Beijing, 100080 China; 4 Puhuangyu community health center, Fengtai District, Beijing, 100079 China

**Keywords:** Coping strategy, Family caregivers, Field study, Home-based palliative care, Need

## Abstract

**Background:**

Most Chinese patients chose to die at home, therefore there is a reliance on the family caregivers to be involved in their palliative care. The needs and coping strategies of family caregivers in home-based palliative care are rooted in culture. Little is known about the needs and coping strategies of family caregivers taking care of dying patients at home.

**Methods:**

A field study using semi-structured interview, participant observation, documents and records collection was employed. The study was conducted in two palliative care outpatient departments in tertiary hospitals and four communities in Beijing, China from March 2021 to July 2022. Using purposive sampling, twenty-five family caregivers were recruited. All collected data were analyzed using content analysis approach.

**Results:**

Five themes emerged, including three care needs and two coping strategies. Family caregivers need to learn care skills and acquire care resources, including (i) decision-making about home-based palliative care, (ii) improving patient’s quality of life, and (iii) signs of final hours and funeral procedures. In facing the care burden, family caregivers coped by (iv) balancing the roles of caregivers and individuals: giving priority to patient care while maintaining their own normal life. In facing the death of a loved one, family caregivers responded by (v) making room for coming death by facing death indirectly and “rescuing” patients for consolation while preparing for the coming death.

**Conclusion:**

Family caregivers strive to balance the roles of being caregivers and being themselves. As caregivers, they actively prepare patients for good death with no regrets. As individuals, they preserve themselves from being hurt to maintain normal life. The needs of family caregivers focus on caregiver role and are manifested in care skills and resources.

**Trial registration:**

Not registered.

**Supplementary Information:**

The online version contains supplementary material available at 10.1186/s12904-023-01315-0.

## Background

Death is very personal for each individual and their family, and personal death and dying in a public place such as a hospital is not ideal for protecting privacy and providing personalized care. Therefore, dying and death at home is considered as a peaceful death [1]. Gomes et al. explored the heterogeneity in preferences for home death through a systematic review which has shown that most patients prefer dying at home, and four-fifths of patients do not change their opinion as their disease progresses [2]. Dying at home provides a chance for patients to maintain their autonomy, privacy, and sense of normality, be surrounded by loved ones, and get physical and emotional support [3]. In China, the most common place of death for adults is at home, accounting for 71.5% [4]. However, another study reported that over 90% of decedents are more likely to be cared in the hospitals for weeks and then be discharged home only a few weeks before death [4]. It implies that it is not common for Chinese people to get care at home.

Home-based palliative care improves patients’ access to care and increases the odds of dying at home [5]. Studies have shown that home-based palliative care can relieve patients’ physical and mental suffering, improve general health, ensure a dignified death, and also reduce psychological distress and burden on family caregivers [6, 7]. Community health centers in Western countries provide home-based palliative care, including risk assessment, symptom management, care guidance, psychological support, and grief counseling, to dying people and their families through family visits, telemedicine, and two-way referrals [8–11]. These home-based palliative care programs improved the quality of life of dying patients and their families and enhanced families’ experience of palliative care when caring for the patient [12]. Experiences from Western countries provide empirical evidence for the development of home-based palliative care in China.

Implementing home-based palliative care in China has been challenging for various reasons. First, no standard home-based palliative care services were developed. Whether to provide home-based palliative care services mainly depends on community health centers in China. Primary healthcare providers working there usually have limited palliative care ability and limited self-efficacy to deliver palliative care actively, and laced multidisciplinary team other than doctors and nurses. Current palliative care service focus on physical care, yet the types of drugs supplied in community health centers are far less than those provided to secondary and tertiary hospitals, hence it might be difficult to effectively control patients’ symptoms [13–16]. Second, it is difficult for community healthcare centers to get support to provide home-based palliative care services. Only 1% of hospitals in China have palliative care institutional services and most of the resources are in tertiary hospitals. What’s more, tertiary hospitals are not required to support community healthcare centers [17]. Third, social support for palliative care is lacking. The professional guidance for home-based palliative care is limited, community volunteer services are fragmented, the medical insurance reimbursement rate for palliative care services is low, and charitable and non-profit organizations are almost non-existent, resulting in insufficient social and institutional support for palliative care at home [18]. Thus, families with dying patients at home are caught on an “isolated island” status in reality.

With the impact of Confucianism, family-oriented culture is prevalent in China, with family members dependent on each other and obligated to care for the sick [19]. Family caregivers are crucial resources in delivering home-based palliative care. However, without enough social support, they face difficulties and challenges when caring for the patients, which resulting in numerous needs [20]. The burden of care and the death of a loved one are also negative stressors for family caregivers, and coping strategies greatly affect their health and well-being [21, 22]. Hence, understanding family caregivers’ needs and coping strategies during the patients’ end-of-life phase is the foundation for professionals to provide tailored support and home-based palliative care to the patient and the family.

Studies in high-ranking palliative care countries, with good palliative care environments, high healthcare affordability, and better community engagement, have reported the needs for home-based palliative care and family caregivers’ coping strategies [23–28]. However, the needs and coping strategies of family caregivers are complex and influenced by various factors, among which culture plays a significant role [29]. Most of these studies come from Western or high-income countries, where the cultural and social background differs from that of China. The purpose of this study is to explore the needs and coping strategies of family caregivers in home-based palliative care in the Chinese cultural context. This study has the potential to inform further development of home-based palliative care programs in China.

## Methods

### Aim

To explore the needs and coping strategies of Chinese family caregivers caring for dying patients at home.

### Study design

In this study, a field study was conducted to understand the needs and coping strategies of family caregivers caring for terminally ill patients in home settings [30]. Field study allows researchers to comprehensively gather information and interpret the process of home-based palliative care through a variety of data collection methods, including interviews, observations, documents and records collections [31]. This study followed the Standards for Reporting Qualitative Research (SRQR) [32].

### Setting

Participants were recruited from two palliative care outpatient departments in tertiary hospitals and four communities in Beijing, China. Tertiary hospitals with palliative care outpatient departments are the main entry points for palliative care services in China. The palliative care outpatient department is responsible for prescribing symptom control medications and providing palliative care information. These two palliative care outpatient departments are national-level demonstrative palliative care bases. The outpatient departments run twice a week, with an average of five family caregivers visiting each time. Four communities were selected where residents can access home-based palliative care services from their community health centers. The directors of these communities, as gatekeepers, assisted the researchers in establishing trust relationships with the participants.

### Participants and recruitment

Purposive sampling was applied to select families of terminally ill patients [33]. The inclusion criteria were adult family caregivers who (a) caring/cared for patients at home for more than three months; (b) caring/cared for patients whose life expectancy of approximately six months or less as estimated by two physicians; (c) were able to provide informed consent. Paid caregivers were excluded.

The first author contacted potential participants through gatekeepers (the directors of two hospitals and four communities). After introducing the purpose of the study and the data would be published anonymously, all caregivers participated in the study and signed a written consent. Informed consent includes the email addresses and phone numbers of the first and corresponding authors, and participants can contact the research team for study details and get support. To make sure the interviews and observations are truthful, the first author build trust and rapport with potential participants in the following ways by (a) introducing identity, interests, and study of researchers at the first meeting, contending with family caregivers, and assisting them with patient care such as turning the patient, washing hair, organizing birthday parties etc., (b) listening patiently to the care process of visiting family caregivers at the outpatient departments to understand their peace and pain, courage and fear, and responding appropriately, and (c) paying return visits to the family caregivers for the visiting problem.

### Data collection

Face-to-face semi-structured interviews were implemented between March 2021 to July 2022 by three trained researchers. Researchers arranged convenient places for participants to conduct interviews. Interviews were recorded using an audio recorder and field notes of interviews were written in a notebook by the researchers. The interview guide was created by first authors and palliative care professors based on the research questions and developed through pre-interviews with 2 participants. It included seven open-ended questions related to the needs and coping strategies of participants caring for dying patients at home (see Supplementary Material). Participants were asked to discuss the content of their care, the support they need to better provide or access palliative care at home, and the coping strategies of family caregivers taking care of dying patients. All interviews were transcribed verbatim in 24 h and transcripts were returned to participants for correction.

Participant observation was used to record family caregivers’ behavior, body language, and words about their needs and coping strategies in home-based palliative care. Based on an observation guideline developed by pre-observation with 2 participants, the first author used a computer to record the observed environment, participant characteristics (gender, relationship to the patient, age), nonverbal behaviors (when, where, and what happened), and verbal interactions (difficulties, needs and coping strategies of palliative care at home, main objectives of outpatient consultation). Immediately after observation, the field notes were collated for recall, and missing details were added. At the same time, reflective journals were kept to record the researcher’s views on the observations, to reduce possible recording bias due to personal characteristics, and to correct errors timely.

To gain an in-depth and accurate understanding of family home care, we also collected documents and records that reflect caregivers’ palliative care needs and coping strategies, such as care logs. The researchers reflected on their own experiences, interests, and preferences before the study so that they could see what impacted their ability to see certain phenomena and conduct this study. Reflections help researchers to realize their abilities and preconceptions, participants’ real experience, and the relationship between the two, which promoted the findings close to the real experience of participants.

### Data analysis

Data were collected and analyzed concurrently. When the data is saturated, we stop collecting data [34]. Data collected by interviews, participant observations, documents and records collection were analyzed using content analysis and managed using the software of Nvivo (11, QSR) [35]. All members of the research team are in the field of palliative care, and all members have qualitative research experience. The first authors read primary data several times, then broke them down into smaller and more manageable meaningful units, followed by coding, creating categories, and grouping them into higher-order themes [36]. The corresponding author provided support and refined the themes. Other researchers provided some suggestions for data analysis. All researchers discussed problems encountered during data analysis weekly to reduce bias. Differences were then identified and discussed until agreement was reached within the team. All transcripts were described, transcribed and analyzed in Chinese, and then translated into English by first authors and corresponding author.

### Ethical considerations

This study was approved by the Ethics Committee (Approval: Z2023SY057) and informed consents were obtained from the participants. As a token of appreciation, each participant received a gift worth 100 RMB (about 14 dollars). Participants have the right to withdraw at any time, and their palliative care services will not be affected, nor will they be held accountable.

## Result

In total, 18 participants were interviewed with an average duration of 70 min (range 33–201 min), 12 of whom received home observation and/or outpatient observation. Seven participants only took part in outpatient observation. The average age of participants was 53.8 years (35 to 80 years), and 64% of them were adult children. Of the 25 participants, more than half were bachelor’s graduates, accounting for 52%, followed by junior high school (24%), senior high school (16%), and master’s degree (8%). Table 1 shows the detailed demographic characteristics of participants.

**Table 1:** Demographic characteristics

Participant	Education	Religion	Diagnosis of Patient	Relationship to Patient	Live with Patient	Type of Data Collection
Family caregiver01	Junior high school	None	Cholangiocarcinoma	Husband	Yes	Interview
Family caregiver02	Bachelor’s degree	None	Chronic myeloid leukemia	Daughter	Yes	Outpatient Observation, Interview
Family caregiver03	Senior high school	None	Lung Cancer	Wife	Yes	Interview
Family caregiver04	Master’s degree	Christianity	Lung Cancer	Husband	Yes	Interview Documents and records collection
Family caregiver05	Junior high school	None	Gastric Cancer	Wife	Yes	Home Observation, Interview
Family caregiver06	Junior high school	None	Gastric Cancer	Daughter	Yes	Interview Documents and records collection
Family caregiver07	Bachelor’s degree	None	Pancreatic Cancer	Son	Yes	Interview
Family caregiver08	Senior high school	None	Liver Cancer	Wife	Yes	Interview Documents and records collection
Family caregiver09	Bachelor’s degree	None	Lung Cancer	Wife	Yes	Outpatient Observation, Interview
Family caregiver10	Bachelor’s degree	None	Alzheimer	Son	No	Outpatient Observation, Interview
Family caregiver11	Bachelor’s degree	None	Lung Cancer	Wife	Yes	Home and Outpatient Observation, Interview
Family caregiver12	Master’s degree	None	Gastric Cancer	Daughter	Yes	Outpatient Observation, Interview
Family caregiver13	Bachelor’s degree	None	Breast Cancer	Son	Yes	Home Observation, Interview
Family caregiver14	Senior high school	None	Alzheimer	Son	No	Home Observation, Interview
Family caregiver15	Senior high school	None	Pancreatic Cancer	Wife	Yes	Outpatient Observation, Interview
Family caregiver16	Bachelor’s degree	None	Colorectal cancer	Daughter	Yes	Home Observation, Interview
Family caregiver17	Bachelor’s degree	None	Ovarian Cancer	Son	Yes	Outpatient Observation, Interview
Family caregiver18	Senior high school	None	Undiagnosed	Son	Yes	Outpatient Observation
Family caregiver19	Bachelor’s degree	None	Lung Cancer	Daughter	Yes	Outpatient Observation
Family caregiver20	Junior high school	None	Gastric Cancer	Husband	Yes	Outpatient Observation, Interview
Family caregiver21	Senior high school	None	Breast Cancer	Son	No	Outpatient Observation
Family caregiver22	Bachelor’s degree	None	Lung Cancer	Daughter	Yes	Outpatient Observation
Family caregiver23	Bachelor’s degree	None	Prostatic Cancer	Son	No	Outpatient Observation
Family caregiver24	Bachelor’s degree	None	Liver Cancer	Daughter	Yes	Outpatient Observation
Family caregiver25	Bachelor’s degree	None	Colon Cancer	Daughter	Yes	Outpatient Observation

**Fig. 1:**
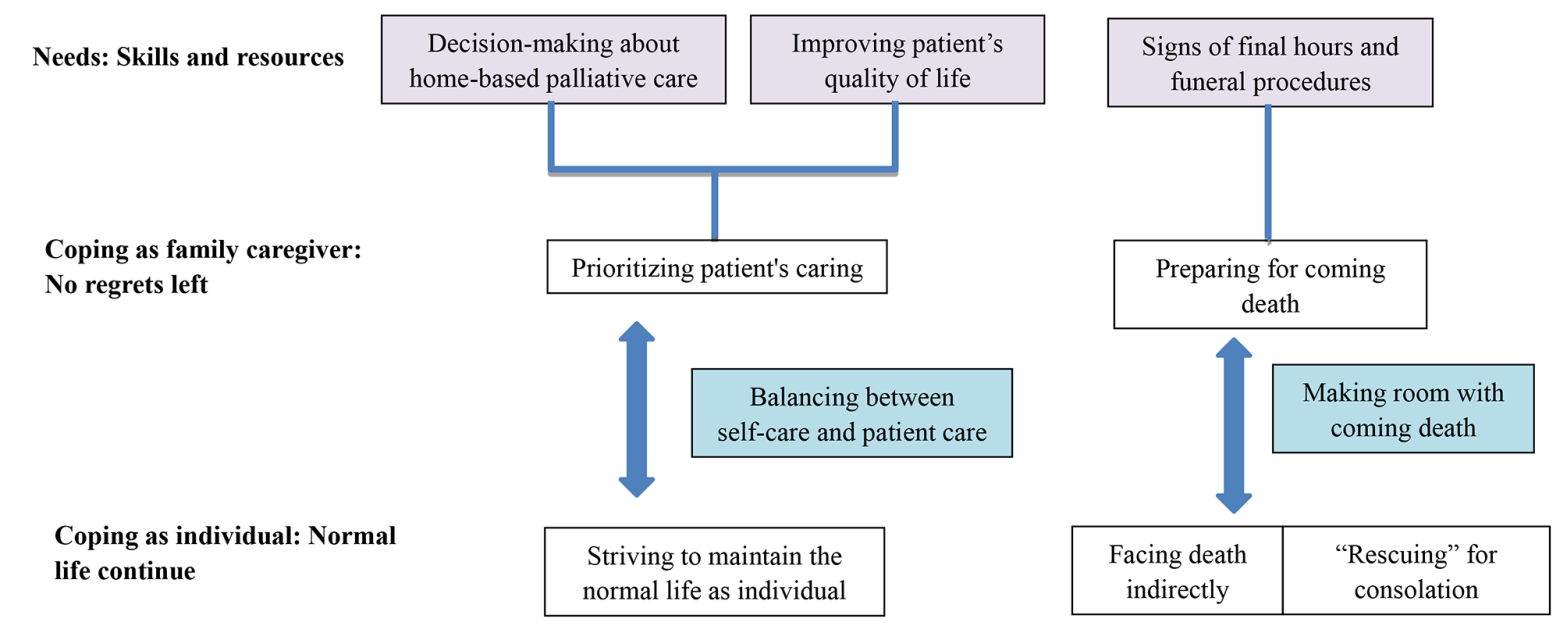
Needs and coping strategies of family caregivers in home-based palliative care

Five themes emerged, including three types of care needs and two coping strategies (see Table 2). Family caregivers play the role of caregiver and individual. As caregivers, they needed to achieve care skills and resources, including decision-making about home-based palliative care, improving patient’s quality of life, signs of final hours, and funeral procedures, in order to leave themselves with no regrets. As individuals, family caregivers also try to being themselves to maintain a normal life. Thus, they coped by balancing between self-care and patient care and facing death indirectly and wisely as a way to give attention to both roles (see Fig. 1).

**Table 2:** Themes and subthemes

Themes	Subthemes
Decision-making about home-based palliative care	Understanding disease progress
	Possibilities and benefits of home-based palliative care
Improving patient’s quality of life	Improving care ability
	Assistance of professionals
Signs of final hours and funeral procedures	Learning about signs of final hours
	Learning about funeral procedures
Balancing between self-care and patient care	Prioritizing patient’s caring
	Striving to maintain the normal life as individual
Making room for coming death	Preparing for impending death
	Facing death indirectly
	“Rescuing” for consolation

### Need 1: decision-making about home-based palliative care

#### Understanding disease progress

Understanding the disease progression helps family caregivers make medical decisions. Family caregiver 18 chose palliative care after confirming with doctors that the patient had no chance of life-saving treatments, “*We decided to stop chemotherapy, which was ineffective and painful for her (patient)*.“ Knowing how long patients have left helps families get confidence in combating the panic caused by the uncertainty of death and helps them to decide where to spend the remaining time at home or in the hospital.

Family caregiver 21 *said he was afraid his mother (patient) would die at home and he couldn’t handle it alone. During the pandemic, hospital policy forbids visitors which means that going to hospital would result in never seeing the patient again. So he would like to know how much time his mother still has.* (Family caregiver 21, Participant observation)

#### Possibilities and benefits of home-based palliative care

Most families were unaware of palliative care. When the treatment effect is minimal, family caregivers expected physicians actively understand the availability and information of palliative care. After family caregiver 04 was advised on home-based palliative care, “*I didn’t know about palliative care before, let alone home-based palliative care. The palliative doctor told us about home-based palliative care. That is what we want*.“

Family caregivers tried to meet the patients’ wish to die at home while considering their caregiving ability. They wanted to know what might happen to the patient in the future and were unsure how to cope. Family caregiver 11, an elderly wife of a person living with lung cancer with bone metastases, was worried about an uncertain future, “*My husband (patient) wanted to die at home, but I don’t know what will happen to him at home. Will I be able to take care of him? I’m afraid I won’t be able to take care of him and aggravate his suffering.*”

Family caregivers’ choice of home-based palliative care depends on whether home-based palliative care can guarantee the quality of life of patients. Family caregivers receive home-based palliative care when they understand that severe and distressing symptoms can be managed.

*After knowing that patient’s dyspnea could be controlled with medication from the doctor*, family caregiver *received home-based palliative care.* (Family caregiver 22, Participant observation)

### Need 2: improving patient’s quality of life

#### Improving care ability

The family caregivers want the patients to live pain-free lives until death, so improving patients’ quality of life is the most important demand. Family caregivers strived to improve their caring abilities, including daily care, symptom recognition, and management. Caring experiences were shared and gained through mutual assistance among family caregivers. Family caregiver 15 mentioned joining a family mutual help WeChat Group is helpful, “*Whenever I have a care problem, I ask in the group, and there is always someone who can give me advice.*” Family caregivers also suggested that community professionals conduct health education lectures.

*I don’t know how to take care of her (patient). I hope that the community health center holds lectures on patient caregiving, such as turning, changing urine pads, preventing pressure sores, maintaining nutrition balance, and preventing constipation, especially after the patient is bedbound.* (Family caregiver 14, Interview)

#### Assistance of professionals

However, it is difficult for family caregivers to deal with all the sufferings of the patients. When family caregivers were unable to cope with the patient’s sufferings, they needed the assistance of professionals. The first response of most family caregivers is to go to a tertiary hospital, “*Tertiary hospitals can provide support that is difficult for patients to access at home.*” (Family caregiver 12, Interview).

What’s more, visits to tertiary hospitals were due to a lack of community palliative care. Family caregiver 05 complained about healthcare services provided by community health centers, “*There are no drugs that can solve cancer pain. Before the pandemic, community nurses were able to come to the home to change catheters, but now this service is not available.”*

Under such realistic conditions, some family caregivers chose to purchase in-home services from For-profit Internet care companies. Family caregiver 02 felt hopeful after learning about paid home care services, “*What I need most now is nursing support at home, even though such services are more expensive than those provided by the community health center and public hospitals.*”

### Need 3: signs of final hours and funeral procedures

#### Learning about signs of final hours

When families realized the patient was running out of time, they had to prepare for the patient’s impending death. Family caregivers who had not experienced death events were nervous about the final moments. Family caregiver 10, whose mother wished to die at home, consulted in advance about the precautions of dying at home, “*What would happen on her (patient) deathbed and what are the signs that she is dying.*”

#### Learning about funeral procedures

Some family caregivers inquired about funeral procedures, focusing mainly on choosing a funeral home, transferring the body, selecting a cemetery, and preparing items that would be used after the patient’s death.

*We haven’t discussed the choice of funeral home and cemetery yet. We don’t know anything about funeral procedures.* (Family caregiver 07, Interview)

### Coping strategy 1: balancing between self-care and patient care

#### Prioritizing patient’s caring

In most cases, family caregivers gave priority to patients’ caring, even if it costs them more money and stress than they can afford. Family caregiver 04 with no income spent a lot of money to satisfy his wife’s wishes, “*I helped her (patient) do whatever she wanted to do, such as buying a house in Hainan and quitting my job to travel around the country with her.*” Some family caregivers kept patients in a good mood, so they tolerated patients’ bad temper and avoided conflict with them. Families also tried to spend as much time with patients as possible during this limited time.

*I spent most of my time with him (patient), and I lost my chance to be with my friends… I’m in a bad mood, but I have to tolerate it.* (Family caregiver 03, Interview)

#### Striving to maintain the normal life as individual

As the disease progressed, family caregivers reported that they could not bear the burden of long-term care alone and needed aid, most likely other family caregivers. Family caregiver 14 has four siblings, but he cared for his mother alone, “*One night I just broke down because of the care stress. At that moment, I thought it was time for my sisters to join me in taking care of my mother (patient).*” Empty-nest families or families with one child often employed paid caregivers to assist with caregiving. Family caregiver 02 is the only child, “*I will be occupied by work for the next period, and I can’t take any more time off, so I have to hire a paid caregiver to take care of my mom (patient).*“

Some of the family caregivers had to give up unbearable and unaffordable care services. Family caregiver 16 developed a lumbar disc herniation from caring for her mother, “*I was unable to care for myself, let alone my mother (patient), so she had to be hospitalized.*” There were also some family caregivers worried about the financial burden of high medical costs.

*Family caregiver 23 said he could not afford to spend 15,000 RMB for a few days in a special palliative care unit. Family caregiver 23 didn’t want the tragedy of spending lots of money with the result of losing the loved one.* (Family caregiver 23, Participant observation)

Family caregivers stated that communicating with others can relieve the distress and pressure of caregiving. They were usually willing to talk to healthcare workers or other family caregivers with similar experiences, *“We have similar care experiences that can resonate… at least, they (other family caregivers who have cared for dying patients) can understand what I’m going through.*” (Family caregiver 13, Interview). We found that family caregivers exuded happiness and contentment after they were recognized by others. Family caregiver 07 recalled taking care of his father, “*My father (patient) was not very conscious, but he always smiled at me when he opened his eyes, which was actually his way of expressing his gratitude to me. All I did was worth it.*”

### Coping strategy 2: making room for coming death

We found that family caregivers were ambivalent when facing death, manifested as preserving themselves by facing death indirectly and “rescuing” patients for consolation while preparing for the coming death. As family caregiver 04 wrote in the care log: *The doctor would hold a family meeting to inform the disease condition and give the doctor’s advice, which included end-of-life arrangements for the patient. It sounds realistic but harsh, and it is still very painful to accept and face.* (Family caregiver 04, Documents and records)

#### Preparing for impending death

Chinese people emphasized the importance of being surrounded by loved ones when the patient is near death. To avoid regret, within days or hours of the patient’s death, the family caregivers notified other family members and friends to visit the patient for a final goodbye.

*I noticed he (patient) was bleeding and his lips were turning white. I thought he was going to die and I sent a message to his sister, asking her to visit him.* (Family caregiver 09, Participant observation)

Family caregivers indicated that they wanted to have enough time with the patient to say their final goodbyes. Some patients and family members with religious beliefs expect a farewell, “*The priest would give a professional farewell and this may comfort us (families).”* (Family caregiver 04, Interview).

Families showed that accepting the impending death of a loved one was quite difficult, but they struggled to cope with the anticipatory grief.

*Family caregiver 25 said every member of her family was having a complicated time accepting her father’s coming death. To ease her grief, she consulted a psychiatrist and took some classes.* (Family caregiver 25, Participant observation)

Some families had changed their views of death as permanent separation. When considering the choice of cemetery, the family caregiver 09 planned to be buried with her husband in the future, “*I bought a multi-burial grave. When I’m gone, we will be together again.*“ When the mother of family caregiver 13 died in his arms, he was at peace, “*When she (patient) died, I would live on with her bloodline. We’ll be together forever.*“

#### Facing death indirectly

Some caregivers were so focused on daily care that they did not want to be mentioned or reminded of the patient’s impending death, which would strain negative emotions. Family caregiver 08 was saddened by the thought of her husband’s terrible condition and did not want to discuss the disease anymore, “*I am a good cook, and my husband (patient) likes to eat the pancakes I make. Now he is so sick that he cannot eat anything. But he insists on not having a feeding tube (pause for a few seconds). I don’t want to talk about it, or I will cry.*”

It was found that family caregivers tacitly refrained from talking about death, even though they had understood the importance of communicating death with patients.

*My mother knew she was terminal, but she never said she was dying. Although we didn’t talk about death, we knew what was probably on each other’s minds. Whenever I made up my mind to talk to her about death, there was always something holding me back, choking me, and making me want to cry.* (Family caregiver 17, Participant observation)

During the interview or daily communication, most family caregivers used euphemisms instead of the words “cancer” or “death”, fearing that these taboo words would irritate patients.

*(Whispering) He (patient) was diagnosed with lung…… disease in 2019 (Looking carefully at the patient, keeping his low voice). He’s awake.* (Family caregiver 01, Interview)

Some family caregivers avoided sensory shocks from the patient’s frightening signs. Family caregiver 24 looked at her mother’s swollen, ball-like stomach, cried and asked, *“What’s wrong? Is there any way to get rid of it?*“ One family caregiver chose ANH (Artificial Nutrition and hydration) even though she knew the patient’s ascites might be related to the high volume of intravenous nutrition.

*Family caregiver 19 explained if her father (patient) was not given intravenous nutrition, he would lose weight and gradually become a skeleton. It would break her heart to see her father like that.* (Family caregiver 19, Participant observation)

#### “Rescuing” for consolation

The study revealed that family caregivers resisted the approach of death and acted to console themselves, whether psychologically prepared or not. Resistance to the approach of death was not intended to prolong the patient’s life, but to bring psychological comfort to the family caregivers. Family caregiver 09 went to the doctor immediately after seeing the patient bleeding and his lips turning white, “*Although I was ready for his (patient) coming death, I still didn’t want that moment to come. Calling a doctor seemed to be a conditioned reflex. I didn’t want him to die because I didn’t do my best.*”

Most families wanted the patient to die peacefully, not suddenly. Therefore, some families arrange for patients to be hospitalized before they die to prevent sudden death.

*If a blockage of sputum makes breathing difficult and an ambulance takes half an hour to arrive at the hospital, it will be too late. If he (patient) died peacefully, I might be ok with that. But if he died with difficulty breathing or in extreme pain, I would be more upset.* (Family caregiver 20, Participant observation)

Faced with the sudden onset of the patient’s potentially fatal symptoms, family caregivers tried their best to “rescue” the patient, even though it was not a medically approved treatment.

*My father (patient) suddenly not feeling well in his heart at home, and my mother was very frightened. She rubbed my father’s palms with the folk medicine, saying that the starting point of pericardium meridian is in the palms, and massaged very hard. My mother did this because she thought she had to do something to stop his condition from worsening.* (Family caregiver 12, Participant observation)

## Discussion

This study explored the needs and coping strategies of family caregivers for home-based palliative care in the context of Chinese death culture using field study. This study found that, in the context of traditional Chinese culture, family caregivers in home-based palliative care presented patient-first care needs and balanced coping strategies. Families give priority to the patients and try their best to care for patients so that they can have a good death. Facing the burden of caregiving and the challenges of death of a loved one, family members strive to balance the roles of caregiver and individual, manifested in seeking a balance between trying to actively prepare patients for good death and protecting themselves as much as possible from the harm caused by the caregiving and death of patients.

This study found that all family caregivers had a basic understanding of palliative care through media coverage or consultations with oncologists, and then they received more information from outpatient palliative care professionals, such as disease progression and illness trajectory. This met the needs of patients and families for palliative information and promoted them to make a smooth transition to palliative care [37]. In addition, our study highlighted the dilemma of family caregivers in making decisions between home and inpatient palliative care. Home-based palliative care means that family caregivers can accompany the patients more often, which is of special significance to the family, but inpatient palliative care means having specialized care and low caregiving burden on the family. Understanding relevant information is a prerequisite for making sound decisions [38]. healthcare providers should be aware of family caregivers’ concerns about the lack of professional support at home, and fully inform family caregivers of the physical, psychological, and spiritual challenges they may face when choosing home-based palliative care.

At the same time, attention should be paid to the paradox of decision-making in home palliative care. First, professionals should focus on continuous ineffective feeding. Food is the paramount necessity of the Chinese people and feeding the patient is a way for family caregivers to show their love. In China, 40.4% of family caregivers usually choose ANH (Artificial Nutrition and hydration), which is mainly performed in hospitals, because they cannot accept that the patient is being starved to death [39]. For patients with ANH that does more harm than good, better education on appropriate ANH should be provided to patients and family caregivers, so as to eliminate the fear of starving patients to death, reduce the struggle of family caregivers between home and hospital, and increase the probability of dying at home. Second, most of the family caregivers in this study were adult children. Filial piety is a social expectation and an important factor in constituting children’s responsibility [40]. Most children, when their parents are ill, especially in the terminal stage, regard the quality of their parents’ life as their own responsibility and make decisions on their parent’s behalf. healthcare workers should address the conflict between family decision-making centered on “filial piety” and respecting patients’ autonomy. In China, family caregivers serve as an important source of information [41], and 84.93% of the decision-making models are dominated by family caregivers [42]. However, there are differences in the type and extent of information needs between patients and caregivers. In order to achieve the dignity of death, decision-making agents are encouraged to communicate more with patients or to make decisions in the best interests of patients.

The study found that family caregivers prioritize patient care and their own lives were disrupted, such as loss of social opportunities, emotional stress, economic pressure, and so on, similar to previous studies [43, 44]. They hide their feelings and needs to portray a “selfless” image and follow the normative guidelines on the “proper” way to behave [45, 46].

In this study, we found that family caregivers always bear the burden and suppress the needs to give priority to patients and maintain normal life of the family. Although family caregivers were aware of the excessive caregiving burden, they continued to suppress their grief and care for patients until the “unbearable” point. They considered seeking help as a “last resort”. This is related to the Chinese character trait of “RenWo” of Chinese people, means Chinese family caregivers tolerate suffering and self-depression for family responsibility, which is a strategic self-restraint mechanism or process in which a person has to do what he or she does not want or suffer what he or she does not want to avoid obviously adverse consequences for himself, others or the public, or to have beneficial consequences [47]. For family caregivers, doing their best to accompany and care for patients is their responsibility and a way to express filial piety [48, 49], but it also causes caregivers to burden themselves with caregiving and become physically and emotionally vulnerable.

The study found that family caregivers are aware of balancing self-care and patient care, but this only occurs when they reach the “unbearable” point because they don’t think “RenWo” is a sign of sacrifice, but a psychological coping strategy to resolve the conflict between ideals and reality. What’s more, it is a positive self-cultivation process to achieve the transcendence of self-will through “RenWo”. Therefore, healthcare workers should pay attention to “RenWo” characteristics and provide them with outlets for emotional release through companionship and listening [50]. Palliative care education can also strengthen family caregivers’ awareness of seeking support in terms of the psychological coping strategies of “RenWo” [51].

The study showed that Chinese family caregivers make room with coming death when facing the death of a loved one. They proactively make behavioral and cognitive preparations for the patient’s impending death, including end-of-life identification at home, coping pathways after death, and preparation for funeral procedures. However, family caregivers strain negative emotions from death during caregiving, which is similar to the ambivalence of anticipatory grief summarized by Coelho and Barbosa [52]. This kind of emotional stress may be related to the family caregiver’s perception of the patient entering the countdown to death. The family caregivers are scratched by the patient’s irreversible state and feel hopeless and helpless. In the face of disorderly times, arranging transactional work, such as immersing in illness care, learning about signs of final hours, and trying to accept the patient’s impending death, maybe the only way to set the order of life rationally [53].

This kind of coping strategy also occurs at the time of death. Although the patients and family caregivers jointly choose home-based palliative care, they subconsciously “rescue” the patient because they could not emotionally accept the patients’ death [54]. As one family caregiver put it, *prolonging the patient’s life will cause him pain, but it will buy time for the living to accept the fact that the patient is going to die* (Family caregiver 08). In addition, the Chinese are deeply influenced by Confucianism, the orthodox Chinese culture, which promotes the concept of “valuing life over death” as the dominant attitude towards death, leading to a universal yearning for longevity. Moreover, filial piety requires family caregivers to respect and comply with the patient’s expectations and to maintain and prolong the patient’s life at all costs [48, 50]. Therefore, family caregivers with insufficient preparedness for death are more likely to ignore the patient’s expectations and deal with the patient’s impending death according to traditional Confucian culture to avoid being blamed or feeling guilty in the future.

Coping with anticipatory grief and preparing emotionally for death can be challenging for families, especially family caregivers, without adequate professional support. As such, emotional coping strategies may be a positive way of self-protection [55, 56]. It is necessary for professionals to understand and recognize family caregivers’ emotional coping strategy for approaching death, and proactively provide tailored support with respect for needs [38, 57, 58]. People who had more communication with patients about end-of-life issues were more likely to report adequate emotional preparation for death and reduced anticipatory grief. However, such communication is considered culturally taboo in China [59, 60]. In this context, professionals should take into account the influence of family’s the sensitivity to the topics of death, assess the willingness and ability to communicate end-of-life issues, and then provide individualized support to facilitate effective end-of-life communication [48, 61, 62].

### Limitations

This study has several limitations. During data collection, we intended to conduct as many household observations as possible. However, community health workers suspended door-to-door services during the epidemic, so it was impossible to observe all participants. In addition, these observation sites were limited to Beijing due to financial constraints. We tried to consider the diversity when selecting the sample. In terms of disease type, only a small number of terminally ill patients with chronic diseases were included, as most of the patients who could be accessed in outpatient departments and in-home services were people living with cancer. Regarding the family-patient relationship, the family caregivers included were mainly the patient’s spouses and children but no parents, which was probably due to the fact that all included patients were adults.

## Conclusion

The study revealed the care needs and coping strategies of family caregivers in home-based palliative care in the context of Chinese culture. Under the influence of Confucianism, family caregivers need care skills and resources to make patient-appropriate decisions, improve patients’ quality of life, and learn about signs of final hours and funeral procedures, in order to leave themselves with no regrets. As individuals, family caregivers also try to preserve themselves in order to maintain a normal life. Thus, they coped by balancing between self-care and patient care and making room for coming death as a way to give attention to both roles. A better understanding of family caregivers’ needs and coping strategies in home-based palliative care could inform healthcare professionals to develop targeted support programs for dying patients and their families and provide detailed information for building a feasible and effective home-based palliative care model in the Chinese context.

### Electronic supplementary material

Below is the link to the electronic supplementary material.

Supplementary Material 1

Supplementary Material 2

## Data Availability

The datasets generated and analyzed during the current study are not publicly available due to confidentiality requirements but are available from the corresponding author on reasonable request.
