# Supplementary material for: Exploring the needs and coping strategies of family caregivers taking care of dying patients at home: a field study
Source: BMC Palliat Care. 2023 Dec 12;22:196. doi: 10.1186/s12904-023-01315-0 (PMC10714660; doi:10.1186/s12904-023-01315-0)
Supplement: Supplementary file 2 — Supplementary Material 2 [file 12904_2023_1315_MOESM2_ESM.docx]

**Interview guideline**

1. Could you please describe your Loved one's disease?

2. What was the reason for choosing home-based palliative care?

3. Did you have any difficulties in caring of patient at home, and if so, what were they?

4. As a family caregiver, how do you cope with these difficulties?

5. What do you think is needed to achieve home-based palliative care?

6.Do you need any support for yourself in care process?

7. Is there anything else you would like to tell me? Or do you have any comments on this interview?
